# Draft Genome Sequence of *Xanthomonas axonopodis* pv. allii Strain CFBP 6369

**DOI:** 10.1128/genomeA.00727-14

**Published:** 2014-07-31

**Authors:** L. Gagnevin, S. Bolot, J. L. Gordon, O. Pruvost, C. Vernière, I. Robène, M. Arlat, L. D. Noël, S. Carrère, M.-A. Jacques, R. Koebnik

**Affiliations:** a CIRAD, UMR Peuplements Végétaux et Bioagresseurs en Milieu Tropical (PVBMT), Saint-Pierre, La Réunion, France; b INRA, Laboratoire des Interactions Plantes Micro-Organismes (LIPM), UMR 441, Castanet-Tolosan, France; c CNRS, Laboratoire des Interactions Plantes Micro-Organismes (LIPM), UMR 2594, Castanet-Tolosan, France; d Université de la Réunion, UMR Peuplements Végétaux et Bioagresseurs en Milieu Tropical (PVBMT), Saint-Pierre, La Réunion, France; e Université de Toulouse, Université Paul Sabatier, Toulouse, France; f INRA, Institut de Recherche en Horticulture et Semences (IRHS) and UMR 1345 SFR 4207 QUASAV, Beaucouzé, France; g Université d’Angers, Institut de Recherche en Horticulture et Semences (IRHS) and UMR 1345 SFR 4207 QUASAV, Beaucouzé, France; h Agrocampus Ouest, Institut de Recherche en Horticulture et Semences (IRHS), UMR 1345 SFR 4207 QUASAV, Beaucouzé, France; i IRD, UMR 186 IRD-CIRAD-Université Montpellier 2 Résistance des Plantes aux Bioagresseurs, Montpellier, France

## Abstract

We report here the draft genome sequence of *Xanthomonas axonopodis* pv. allii strain CFBP 6369, the causal agent of bacterial blight of onion. The draft genome has a size of 5,425,942 bp and a G+C content of 64.4%.

## GENOME ANNOUNCEMENT

*Xanthomonas axonopodis* pv. allii is the causal agent of bacterial blight of onion (*Allium cepa*) and other *Allium* species (1, 2). First reported in 1978 (3), the disease has been emerging since the 1990s. It is included in the A1 list of pathogens of the European and Mediterranean Plant Protection Organization (EPPO). Lesions on leaves infected with this pathogen begin as small water-soaked spots that enlarge into chlorotic lesions and collapse at the point of initial infection, causing a reduction in bulb size. The bacterium can contaminate seeds, which are a major source of inoculum (4, 5), together with diseased plants and plant debris, epiphytically polluted *Allium* or weed species, and irrigation water. Seed trade is responsible for long-distance dissemination (6, 7). Yield losses can reach 50% and are favored by high temperature and humidity. The control of the disease can be partially achieved through integrated pest management (8, 9) and should improve with better knowledge of the bacterium and its interaction with its host species. *X. axonopodis* pv. allii strains are more genetically diversified and less host specialized than is usually expected for a *Xanthomonas* pathovar. They belong to the genetically diverse group 9.2 of *X. axonopodis* (1) and are probably polyphyletic (10). Strain CFBP 6369, isolated in 1996 in Réunion Island, France, was chosen because its genetic, physiologic, and pathogenic characteristics are representative of the pathovar (1).

Strain CFBP 6369 was sequenced using the Illumina HiSeq 2000 platform (GATC Biotech, Germany). The shotgun sequencing yielded 35,091,791 read pairs (21,359,123 100-bp paired-end reads with an insert size of ca. 250 bp, and 13,732,668 50-bp mate-pair reads with an insert size of ca. 3 kb). A combination of Velvet (11), SOAP*denovo*, and SOAPGapCloser (12) yielded 13 contigs >500 bp (*N*_50_, 1,415,587 bp), with the largest contig being 1,460,767 bp, for a total assembly size of 5,425,942 bp. The G+C content was 64.4%. Genomic contigs were annotated using the EuGene-P annotation pipeline to identify RNAs and protein-coding genes (13). The draft genome of strain CFBP 6369 was predicted to contain 4,963 coding sequences (CDSs).

The genome of strain CFBP 6369 has an average nucleotide identity (ANI) close to 99% (14) with other xanthomonads in the 9.2 group, while it shares <95% ANI with strains from groups 9.5 and 9.6. *X. axonopodis* pv. allii has a gene content similar to those of other xanthomonads, with a large representation of two-component system sensor and regulatory proteins, methyl-accepting chemotaxis proteins, ATP-binding cassette (ABC) transporters, major facilitator superfamily (MFS) transporters, TonB-dependent receptors, and transcriptional regulators. It contains a complete set of genes coding for the hypersensitive response and pathogenicity (HRP) type III secretion system and a minimal repertoire of 22 putative type III effector genes: *avrBs2*, *xopA*, *xopAD*, *xopAE*, *xopAJ*, *xopAK*, *xopC2*, *xopE1*, *xopF1*, *xopF2*, *xopI*, *xopJ*, *xopK*, *xopL*, *xopN*, *xopP*, *xopQ*, *xopR*, *xopV*, *xopX*, *xopZ1*, and *xopAP*. This resource will be instrumental for dissecting the genetic and molecular basis of *X. axonopodis* pv. allii pathogenicity on *Alliaceae*, investigating the natural genomic diversity of this emerging plant pathogen, and improving diagnostics.

### Nucleotide sequence accession numbers.

This whole-genome shotgun project has been deposited in GenBank under the accession no. JOJQ00000000. The version described in this paper is the first version, JOJQ01000000.

## References

[1] Roumagnac P Gagnevin L Gardan L Sutra L Manceau C Dickstein ER Jones JB Rott P Pruvost O 2004 Polyphasic characterization of xanthomonads isolated from onion, garlic and Welsh onion (*Allium* spp.) and their relatedness to different *Xanthomonas* species. Int. J. Syst. Evol. Microbiol. 54:15–24. 10.1099/ijs.0.02714-0 14742454

[2] Gent DH Schwartz HF Ishimaru CA Louws FJ Cramer RA Lawrence CB 2004 Polyphasic characterization of *Xanthomonas* strains from onion. Phytopathology 94:184–195. 10.1094/PHYTO.2004.94.2.184 18943542

[3] Alvarez AM Buddenhagen IW Buddenhagen ES Domen HY 1978 Bacterial blight of onion, a new disease caused by *Xanthomonas* sp. Phytopathology 68:1132–1136. 10.1094/Phyto-68-1132

[4] Picard Y Roumagnac P Legrand D Humeau L Robène-Soustrade I Chiroleu F Gagnevin L Pruvost O 2008 Polyphasic characterization of *Xanthomonas axonopodis* pv. *allii* associated with outbreaks of bacterial blight on three *Allium* species in the Mascarene archipelago. Phytopathology 98:919–925. 10.1094/PHYTO-98-8-0919 18943210

[5] Roumagnac P Gagnevin L Pruvost O 2000 Detection of *Xanthomonas* sp., the causal agent of onion bacterial blight, in onion seeds using a newly developed semi-selective isolation medium. Eur. J. Plant Pathol. 106:867–877. 10.1023/A:1008743120242

[6] Gent DH Lang JM Bartolo ME Schwartz HR 2005 Inoculum sources and survival of *Xanthomonas axonopodis* pv. *allii* in Colorado. Plant Dis. 89:507–514. 10.1094/PD-89-0507 30795430

[7] Gent DH Lang JM Schwartz HF 2005 Epiphytic survival of *Xanthomonas axonopodis* pv. *allii* and *X. axonopodis* pv. *phaseoli* on leguminous hosts and onion. Plant Dis. 89:558–564. 10.1094/PD-89-0558 30795378

[8] Lang JM Gent DH Schwartz HF 2007 Management of *Xanthomonas* leaf blight of onion with bacteriophages and a plant activator. Plant Dis. 91:871–878. 10.1094/PDIS-91-7-0871 30780399

[9] Robène-Soustrade I Legrand D Gagnevin L Chiroleu F Laurent A Pruvost O 2010 Multiplex nested PCR for detection of *Xanthomonas axonopodis* pv. *allii* from onion seeds. Appl. Environ. Microbiol. 76:2697–2703. 10.1128/AEM.02697-09 20208024 PMC2863456

[10] Gent DH Al-Saadi A Gabriel DW Louws FJ Ishimaru CA Schwartz HF 2005 Pathogenic and genetic relatedness among *Xanthomonas axonopodis* pv. *allii* and other pathovars of *X. axonopodis*. Phytopathology 95:918–925. 10.1094/PHYTO-95-0918 18944414

[11] Zerbino DR Birney E 2008 Velvet: algorithms for *de novo* short read assembly using de Bruijn graphs. Genome Res. 18:821–829. 10.1101/gr.074492.107 18349386 PMC2336801

[12] Luo R Liu B Xie Y Li Z Huang W Yuan J He G Chen Y Pan Q Liu Y Tang J Wu G Zhang H Shi Y Liu Y Yu C Wang B Lu Y Han C Cheung DW Yiu SM Peng S Xiaoqian Z Liu G Liao X Li Y Yang H Wang J Lam TW Wang J 2012 SOAP*denovo*2: an empirically improved memory-efficient short-read *de novo* assembler. GigaScience. 1:18. 10.1186/2047-217X-1-18 23587118 PMC3626529

[13] Sallet E Roux B Sauviac L Jardinaud MF Carrère S Faraut T de Carvalho-Niebel F Gouzy J Gamas P Capela D Bruand C Schiex T 2013 Next-generation annotation of prokaryotic genomes with EuGene-P: application to *Sinorhizobium meliloti* 2011. DNA Res. 20:339–354. 10.1093/dnares/dst014 23599422 PMC3738161

[14] Goris J Konstantinidis KT Klappenbach JA Coenye T Vandamme P Tiedje JM 2007 DNA-DNA hybridization values and their relationship to whole-genome sequence similarities. Int. J. Syst. Evol. Microbiol. 57:81–91. 10.1099/ijs.0.64483-0 17220447

